# Epigenetic consequences of artificial reproductive technologies to the bovine imprinted genes *SNRPN*, *H19/IGF2*, and *IGF2R*

**DOI:** 10.3389/fgene.2015.00058

**Published:** 2015-02-25

**Authors:** Lawrence C. Smith, Jacinthe Therrien, France Filion, Fabiana Bressan, Flávio V. Meirelles

**Affiliations:** ^1^Department of Veterinary Biomedicine, Centre de Recherche en Reproduction Animale, Faculty of Veterinary Medicine, University of Montreal, Saint-Hyacinthe, QC, Canada; ^2^Department of Veterinary Medicine, Faculty of Animal Sciences and Food Engineering, University of São Paulo, Pirassununga, Brazil

**Keywords:** epigenetics, genomic imprinting, *in vitro* culture, nuclear transfer, animal cloning, cattle

## Abstract

Animal breeders have made widespread use of assisted reproductive technologies to accelerate genetic improvement programs aimed at obtaining more, better and cheaper food products. Selection approaches have traditionally focused on Mendel’s laws of inheritance using parental phenotypic characteristics and quantitative genetics approaches to choose the best parents for the next generation, regardless of their gender. However, apart from contributing DNA sequence variants, male and female gametes carry parental-specific epigenetic marks that play key roles during pre- and post-natal development and growth of the offspring. We herein review the epigenetic anomalies that are associated with artificial reproductive technologies in current use in animal breeding programs. For instance, we demonstrate that bovine embryos and fetuses derived by *in vitro* culture and somatic cell nuclear transfer show epigenetic anomalies in the differentially methylated regions controlling the expression of some imprinted genes. Although these genomic imprinting errors are undetected in the somatic tissues after birth, further research is warranted to examine potential germ cell transmission of epimutations and the potential risks of reproducing cattle using artificial reproductive technologies.

## INTRODUCTION

Artificial reproductive technologies (ART) have been widely applied to improve the fertility of domestic species, particularly in bovine livestock production systems. From the less invasive ART of artificial insemination using frozen semen to the most recent advances in animal cloning by somatic cell nuclear transfer (SCNT), few studies have focused on the potential long term consequences, both positive and negative, of reproductive technologies to domestic animal health and welfare (Urrego et al., 2014). Moreover, scientific evidence for transgenerational inheritance of features acquired from the environment during lifetime, e.g., food intake, is mounting and it is possible that such epigenetic phenomena could affect animal production and reproduction traits for several generations. Examples of epigenetic inheritance induced by genetic (Morgan et al., 1999; Grandjean et al., 2009) and environmental (Ng et al., 2010) perturbations have been shown in rodents, and the mechanisms involved have begun to be elucidated (Daxinger and Whitelaw, 2012; Heard and Martienssen, 2014). Furthermore, in humans, retrospective cohort studies demonstrate transgenerational effects of prenatal famine exposure on birth weight and metabolic disease rates (Pembrey et al., 2006; Painter et al., 2008; Lumey et al., 2009), suggesting that epigenetic modifications caused by metabolic changes to domestic animals may also be transgenerationally inherited.

The role of epigenetics and, particularly of imprinted genes, in domestic animal production traits remains largely unknown. According to recent information obtained on the Geneimprint website (www.geneimprint.org), which provides a listing of genes by species, only a few imprinted genes have been identified in domestic species (cow, *n* = 19; sheep, *n* = 17; pig, *n* = 22) compared to the much larger number already identified in humans (*n* = 95) and mice (*n* = 123). Nonetheless, imprinted genes have been found in livestock to affect traits such as milk yield, growth and carcass quality, fat and meat deposition and fetal development. For instance, the paternally expressed imprinted gene insulin-like growth factor 2 (*IGF2*) has been associated with meat traits and body weight in beef cattle (Flisikowski et al., 2007; Goodall and Schmutz, 2007; Sherman et al., 2008; Bagnicka et al., 2010), and muscle mass and fat deposition in pigs (Van Laere et al., 2003; Vykoukalova et al., 2006; Stinckens et al., 2010). Considering the important role *IGF2* and other imprinted genes have on a number of production traits, it is likely that the inclusion of genomic imprinting in the calculation of breeding values using traditional phenotypic records (pedigree-based selection) and the more recent use of DNA polymorphisms (genomic selection) will be useful to improve animal selection schemes, as indicated by timely recent review articles on this topic (Goddard and Whitelaw, 2014; Magee et al., 2014).

Animal cloning by SCNT has highlighted the importance of epigenetics on the phenotype. Although clones carry genetically identical chromosomes, wide-ranging phenotypic variations have been identified in a number of growth parameters, particularly during prenatal stages of life, when epigenetic errors can lead to the aberrant expression of genes that play a key role in the development and differentiation of both embryonic and extra-embryonic tissues. Nonetheless, high variability of body size has been observed in bovine clones derived by nuclear transfer, suggesting that epigenetic errors may also play a role after birth (Gärtner et al., 1998; Heyman et al., 2004). Since cloned animals carry epimutations to a number of imprinted genes, it is possible that such epigenetic marks could be transmitted through their germ cells to the following generations.

## DEVELOPMENTAL ANOMALIES OBSERVED IN ANIMAL CLONING ARE SIMILAR TO THOSE OBSERVED IN GESTATIONS FROM *IN VITRO* PRODUCED EMBRYOS

The low survival and high morbidity of cloned offspring has become a major obstacle for a wide scale commercial implementation of the SCNT methodology (Smith et al., 2012). The Food and Drug Agency of the USA government has compiled thousands of data from cloned cows, pigs and goat to conclude that the food products from cloned animals and from their offspring are not different from naturally bred animals and, therefore, carry no health risk for human consumption. Nonetheless, public perception has rendered consumers reticent and few retailers are willing to introduce cloned animal products in the food chain. Phenotypic abnormalities associated with somatic cell cloning have been described in detail by numerous laboratories worldwide (Heyman et al., 2002; Wells et al., 2004), including recent reports describing the various neonatal clinical outcomes observed in cloned progeny of both *Bos taurus* (Brisville et al., 2011; Buczinski et al., 2011; Kohan-Ghadr et al., 2011) and *Bos indicus* cattle (Meirelles et al., 2010).

## EPIGENETIC REPROGRAMMING OF GENOMIC IMPRINTS DURING GAMETOGENESIS AND EARLY EMBRYONIC DEVELOPMENT

Epigenetics is the hereditary variation in genomic activity that is independent of any alteration of the DNA sequence. Epigenetic inheritance refers to the memory of such activity; transferred between cellular generations through mitosis, and between organismal generations through meiosis and, therefore, is a link between genotype and phenotype that controls the expression of a locus. Several different types of epigenetic modifications contribute to stabilize gene expression in specialized cell types so that cellular identity and lineage fidelity is preserved, including those that alter chromatin structure, modify DNA and histones, remodel nucleosomes and incorporate variant histones. However, epigenetic stability is reprogrammed globally in two phases of the life cycle: in primordial germ cells (PGC) during gametogenesis and then in the zygote immediately after fertilization. The widespread epigenetic reprogramming in PGCs is associated with a global demethylation and is thought to be important for preventing the transmission of inappropriate epigenetic information to the next generation. Although zygotic demethylation seems to occur globally, some genomic sequences, including imprinted genes, maintain methylation during preimplantation and are essential for proper development. Moreover, some histones are retained at non-random genomic locations in the sperm (Wykes and Krawetz, 2003) and can be inherited in the zygote (van der Heijden et al., 2008), thus allowing that the protection of DNA methylation at some imprints enables the inheritance of histone modifications from the gametes to the next generation.

Genomic imprinting is defined as a process by which certain genes are expressed differentially according to the parent-of-origin and is, therefore, independent of the classical Mendelian inheritance model. Both the establishment and the maintenance of genomic imprints during embryogenesis are essential for the proper embryonic and placental development. For instance, the imprinted gene *H19* (non-protein coding) is silenced on the paternal allele, thus enabling *IGF2* paternal expression. On the other hand, *H19* is expressed from the non-silenced maternal allele where it blocks *IGF2* maternal expression. In contrast, the small nuclear ribonucleaoprotein polypeptide (*SNRPN*) gene is silenced on the maternal allele and, thus, is monoallelically expressed from the paternal allele. Moreover, the receptor for *IGF2* (*IGF2R*) is transcribed exclusively from the maternal allele due to the repression of paternal transcription by the non-coding *AIR* transcript. The epigenetic processes of DNA methylation and histone modulation are both involved in achieving monoallelic expression of imprinted genes. The numerous human genetic diseases associated with imprinted genes, including Beckwith–Wiedemann syndrome (BWS; Brown et al., 1996), Silver–Russel syndrome (Bartholdi et al., 2009), Angelman syndrome (Runte et al., 2004) and Prader–Willi syndrome (Reed and Leff, 1994), evidence the importance of allele-specific expression patterns of imprinted genes. Interestingly, risks for some of the above syndromes are elevated in children conceived by ART (Maher et al., 2003), suggesting that the exposure of gametes and/or early embryos to *in vitro* environments may cause epigenetic alterations to imprinted genes.

## EPIGENETIC REPROGRAMMING OF GENOMIC IMPRINTS IN CLONED EMBRYOS AND FETUSES

A common condition in ruminants derived from ARTs is the large offspring syndrome, or LOS (Young et al., 1998; Ceelen and Vermeiden, 2001), which correlates with *IGF2R* imprinting disruption (Young et al., 2001) and is considered as reminiscent of and therefore a suitable model for the BWS in humans (Chen et al., 2013). As described above (Developmental anomalies observed in animal cloning are similar to those observed in gestations from *in vitro* produced embryos), LOS commonly presents placental perturbations, leading to high birth weights and reduced survival rates. The incidence of such placental failures is especially important in cloning by nuclear transfer and IVF, representing the major cause of pregnancy failure in these animals (Heyman et al., 2002; Hill, 2002; Constant et al., 2006).

**FIGURE 1:**
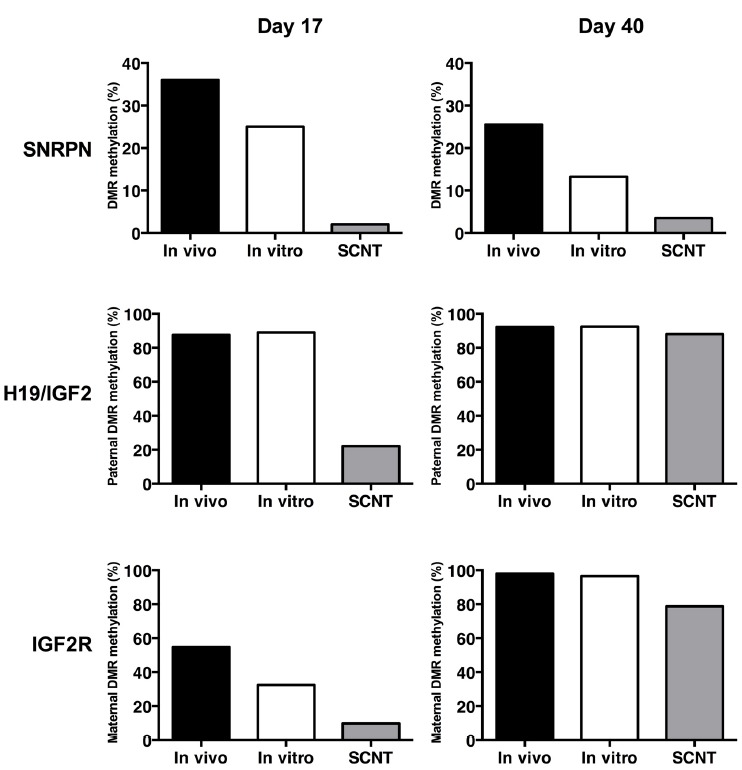
**DNA methylation levels of the DMRs from bovine imprinted genes *SNRPN*, *H19/IGF2*, and *IGF2R* in embryos (day 17) and early foetuses (day 40) derived *in vivo* (back bar), *in vitro* (white bar) and by SCNT (grey bar)**.

We have identified the DMRs of a series of imprinted genes that could potentially be implicated with placental development and the LOS phenotype in cattle. Using a bovine F1 hybrid model (*Bos taurus* vs. *Bos indicus*), we were able to identify SNPs in proximity to the DMR and exonic polymorphisms that enabled analysis of the imprinting status of several genes, including the maternally expressed *H19* and *IGF2R* genes, and the paternally expressed *SNRPN* gene. Analysis was performed on day-17 pre-implantation embryos, in post-implantation embryonic and extra-embryonic (placenta) tissues at around day 40 of gestation and in full term offspring. In general, we observe a de-methylation of the DMR at early embryonic stages (day 17) of development followed by a partial or complete re-methylation of the affected DMR of all three imprinted genes at the fetal stages (day 40–50) of development (Figure 1).

### REPROGRAMMING OF THE *SNRPN* GENOMIC IMPRINT

Methylation patterns of the *SNRPN* DMR are affected in SCNT, whereas no differences were observed between *in vivo*- and *in vitro*-derived day 17 embryos (Lucifero et al., 2006), suggesting that this epigenetic anomaly is caused by an erroneous reprogramming of the somatic cell chromatin by the oocyte cytoplasm and not by the culture conditions employed during initial development to the blastocyst stage. In follow-up experiments we confirmed that the *SNRPN* DMR demethylation of SCNT is also observed after implantation, in both embryonic (brain, heart, liver, and skeletal muscle) and extra-embryonic (placenta) tissues (Suzuki et al., 2009). Surprisingly, a less prominent hypomethylation of the *SNRPN* DMR was also observed in *in vitro*-derived embryonic and extraembryonic tissues, indicating that *in vitro* culture during early development can induce epigenetic errors that persist beyond implantation. Indeed, having identified a *Bos indicus* SNP within exon 2 of *SNRPN*, we demonstrated that whereas *in vivo*-derived day-17 are mono-allelic, i.e., expressing exclusively from paternal allele, both *in vitro* and SCNT embryos showed significantly higher levels of maternal expression. However, *in vitro*-derived bi-allelic expression was no longer observed in embryonic tissues after implantation (day 45), suggesting that the hypomethylation patterns observed in these tissues are insufficient to induce maternal expression. Nonetheless, placental tissues remained bi-allelic beyond implantation, indicating that the extraembryonic tissue may sustain higher levels of hypomethylation. In contrast, SCNT-derived embryonic and extra-embryonic tissues showed substantial bi-allelic expression and DMR hypomethylation, indicating that even embryos with severe epigenetic anomalies to *SNRPN* can survive beyond implantation.

### REPROGRAMMING OF THE *H19/IGF2* GENOMIC IMPRINT

To extend our finding to other bovine imprinted loci, we identified SNPs in the DMR and the transcript (cDNA) of the *H19* gene in cattle (Suzuki et al., 2011). Analysis of the DMR of the maternally expressed *H19* gene indicated a hypermethylation of the paternal allele in the CTCF binding site. Similar to *SNRPN*, SCNT caused the hypomethylation of the imprinted paternal allele in day 17 pre-implantation embryos, which correlates significantly with increased expression of *H19* from the paternal allele, i.e., bi-allelic expression. Interestingly, compared to the *in vivo* group, the overall transcript abundance level of both *H19* and *IGF2* was reduced in SCNT and *in vitro*-derived embryos. During post-implantation, methylation of the *H19* CTCF was not affected in placenta, but was reduced in SCNT embryonic muscle when compared to *in vivo* and *in vitro* samples. However, in contrast to the unaffected *SNRPN* DMR during post-implantation, SCNT led to a partial hypomethylation of the paternal DMR and CTCF binding site on the paternally imprinted allele. Nonetheless, exclusive mono-allelic maternal expression was observed in both embryonic (muscle) and extra-embryonic (placenta) post-implantation tissues, indicating that the hypomethylation levels were insufficient to enable expression from the paternal allele. Interestingly, overall transcript abundance levels of *IGF2*, but not *H19*, were slightly increased in the placenta of SCNT-derived gestations. Since SCNT embryos are usually smaller, it is possible that increased *IGF2* expression adversely affects the growth rate of the embryo itself.

### REPROGRAMMING OF THE IGF2R GENOMIC IMPRINT

The DMR2 of the bovine *IGF2R* gene is between 2 to 2.7 kb in length and is localized in the second intron, approximately 4.4 kb upstream from exon 2 and 2.2 kb downstream from exon 3 (Smith et al., 2012). As expected, the DMR is non-methylated in sperm DNA and hypermethylated in oocytes. With the identification of SNPs in the *Bos indicus* cattle, we have been able to confirm that the *IGF2R* DMR in somatic tissues of adult F1 individuals shows hypomethylation of the paternal allele and hypermethylation of the maternal allele. Preliminary analysis of the paternally imprinted *IGF2R* gene in day 17 embryos indicates that, although the methylation patterns of the DMR are reduced in the SCNT, expression is consistently bi-allelic, regardless of whether the embryos are derived *in vivo*, *in vitro* or by SCNT. At day 45 of development, the *IGF2R* DMR in fetal tissues is consistently hypermethylated whereas the placenta is significantly less methylated, particularly in the SCNT group. Nonetheless, *IGF2R* biallelic expression is present in all tissues, regardless of the method employed to derive the embryos, i.e., *in vivo*, *in vitro*, or SCNT. Therefore, *IGF2R* seems not to be strongly imprinted at any stage in development in cattle, indicating that an epigenetic mechanism other than the methylation of the DMR may be implicated in controlling allele-specific expression.

## EPIGENETIC REPROGRAMMING OF GENOMIC IMPRINTS IN POSTNATAL CLONES

Abnormal methylation patterns have been observed in cloned ruminants, and these epigenetic anomalies have been associated with developmental effects that lead to postnatal mortality, i.e., the LOS syndrome (Young et al., 2003; Curchoe et al., 2009). To further examine whether LOS derived bovine clones show imprinted gene defects using our allele-specific analysis, we analyzed the paternal and maternal methylation patterns of the *H19* and *IGF2R* DMRs and expression patterns. In contrast to previous reports, adult somatic tissues of cloned calves that died within 24 h after birth showed normal levels of methylation relative to healthy clones and to animals born by artificial insemination. Moreover, maternal *IGF2R* DMRs in tissues from *in vivo* and cloned individuals are consistently hypermethylated, regardless of whether the clones were born in good health. However, levels of *IGF2R* bi-allelic expression proved to be higher in *in vivo*-derived than clones. Together, these results indicate that SCNT-derived animals that develop to term show normal methylation of the *H19* imprinted locus, regardless of their health condition at birth.

## EPIGENETIC REPROGRAMMING OF GENOMIC IMPRINTS IN INDUCED PLURIPOTENT STEM CELLS

**FIGURE 2:**
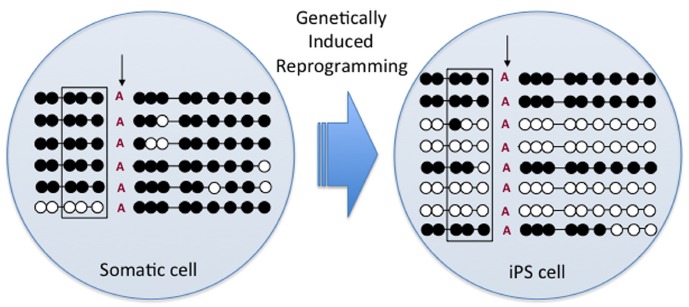
**DNA methylation pattern of the paternal *H19/IGF2* DMR in hybrid fibroblasts (somatic cell) and induced pluripotent stem (iPS) cells.** Circles represent methylated (black) and non-methylated (white) CpGs on the paternal allele DMR as confirmed by a *Bos indicus*-specific SNP (vertical arrow). Square indicates location of the CTCF binding site also indicating significant de-methylation after iPS cell reprogramming.

Apart from SCNT, reversion to pluripotency in somatic cells has been achieved through the induction with pluripotency-related factors, resulting in induced pluripotent stem (iPS) cells. Recent preliminary studies with bovine iPS cell lines developed from F1 hybrid (*B. taurus* × *B. indicus*) fetal fibroblasts using transduction with a polycistronic excisable lentivirus containing mouse *Oct4*, *Sox2 c-Myc*, and *Klf-4* transcription factors have indicated the *H19* and *SNRPN* DMRs are hypomethylated in some iPS lines (Figure 2; Bressan et al., 2014). Moreover, gene expression analyses revealed a bi-allelic expression of *H19* and decreased global expression of both *H19* and *IGF2* in most iPS lines. However, *SNRPN* transcripts were exclusively monoallelic regardless of a significant increase in global expression of *SNRPN*. Together, these preliminary results indicate that genetically induced reprogramming of somatic cells can lead to epigenetic alterations to bovine imprinted genes in a manner similar to SCNT and *in vitro* embryo production, supporting the use of iPS cells as a model system to analyze the effects of ART on imprinted genes.

## CONCLUSIONS AND FUTURE PERSPECTIVES

In general, DMRs of *SNRPN*, *H10/IGF2*, and *IGF2R* imprinted genes are extensively hypomethylated in the early stage embryos derived by SCNT and to a lesser extent also in IVP embryos, indicating that the reprogramming of the chromatin and the culture *in vitro* of oocytes and or embryos causes epigenetic erasure of imprinted loci. Moreover, fewer epigenetic anomalies are observed at later stages of development, suggesting that the imprinted DMRs are either re-methylated during development or that only the less affected embryos survive to later stages of gestation and to term.

Epigenetic errors are likely to have significant effects on the prenatal and postnatal phenotype of domestic animals. Strategies aiming to modify the epigenome by promoting or inhibiting methylation/acetylation drugs prior or during SCNT have been used, however, results remain inconsistent (Sangalli et al., 2014). Since evidence is mounting that show some epigenetic errors result from handling gametes and early embryos *in vitro*, particularly with cloning by SCNT, further care must be taken when utilizing ART to improve the reproductive performance or accelerate the dissemination of valuable livestock genetic material. However, animals that develop to term seem to retain normal methylation patterns of regulatory regions that control the expression of imprinted genes regardless of their health status, suggesting that embryos carrying harmful epimutations may be selectively eliminated before birth. On the other hand, epigenetic variants that improve survival could be useful for generating animals with increased efficiency. Finally, epigenetic modifications to animals derived by ART could bring transgenerational advantages and risks to animal breeding programs.

### Conflict of Interest Statement

The authors declare that the research was conducted in the absence of any commercial or financial relationships that could be construed as a potential conflict of interest.
